# N- and C-Modified TiO_2_ Nanotube Arrays: Enhanced Photoelectrochemical Properties and Effect of Nanotubes Length on Photoconversion Efficiency

**DOI:** 10.3390/nano8040198

**Published:** 2018-03-28

**Authors:** Ahmed El Ruby Mohamed, Shahzad Barghi, Sohrab Rohani

**Affiliations:** 1 Department of Chemical and Biochemical Engineering, Western University, London, ON N6A 5B9, Canada; sbarghi2@uwo.ca; 2 Department of Advanced Materials, Central Metallurgical Research and Development Institute (CMRDI), P.O. Box 87, Helwan, Cairo 11421, Egypt

**Keywords:** TiO_2_ nanotube arrays, N- and C-modification, band gap, recombination rate, photocurrent, photoconversion efficiency

## Abstract

In this investigation, a new, facile, low cost and environmental-friendly method was introduced to fabricate N- and C-modified TiO_2_ nanotube arrays by immersing the as-anodized TiO_2_ nanotube arrays (TNTAs) in a urea aqueous solution with mechanical agitation for a short time and keeping the TNTAs immersed in the solution for 6 h at room temperature. Then, the TNTAs were annealed at different temperatures. The produced N-, C-modified TNTAs were characterized using FESEM, EDX, XRD, XPS, UV-Vis diffuse reflectance spectra. Modified optical properties with narrow band gap energy, E_g_, of 2.65 eV was obtained after annealing the modified TNTAs at 550 °C. Modified TNTAs showed enhanced photoelectochemical performance. Photoconversion efficiency (PCE) was increased from 4.35% for pristine (unmodified) TNTAs to 5.18% for modified TNTAs, an increase of 19%. Effect of nanotubes length of modified TNTAs on photoelectrochemical performance was also studied. Photocurrent density and PCE were increased by increasing nanotube length with a maximum PCE of 6.38% for nanotube length of 55 µm. This high PCE value was attributed to: band gap reduction due to C- and N-modification of TNTAs surface, increased surface area of long TNTAs compared with short TNTAs, investigated in previous studies.

## 1. Introduction

During the last fifteen years, and after the first work of Zwilling et al. [1], titania nanotube arrays (TNTAs) fabricated by electrochemical anodization method have attracted a great deal of attention of scientists, engineers and technologists all over the world due to their outstanding properties such as high specific surface area [2], high charge transport and separation rates [3], light absorption and propagation properties [4,5,6], biocompatibility and chemical stability [7]. Furthermore, the integrated, vertically oriented, highly ordered nanotubular structure strongly adhered to Ti parent metal substrate imparts a very important practical advantage by eliminating the costly solid/liquid separation step which is necessary when using the nanoparticles counterpart. Due to these properties, TNTAs have proved to be a promising candidate in many advanced applications including dye-sensitized solar cells [8,9,10,11,12], sensors development [13,14], hydrogen generation from water by photoelectrolysis [15,16,17,18,19], photocatalysis [20,21,22,23], self-cleaning [24], bone and medicinal implants [25], drug delivery and biomaterials applications [26], and molecular filtration [27]. In the last few years, many interesting advanced applications of TNTAs have been reported. For instance, Bella et al. employed self-standing TNTAs grown on Ti- mesh as a photoanode and photoinduced polymerized polymer electrolyte to fabricate a quasi-solid flexible dye-sensitized solar cell for portable devices. The photopolymerization resulted in a crosslinked polymer electrolyte membrane as the photoanode and the separating electrolyte to prevent the short cut of the solar cell [28]. Marien et al. investigated the influence of TNTAs length on the photodegradation of paraquat. The photocatalytic activity of TNTAs was increased by increasing the nanotube length and reached the optimal value at 7 µm nanotube length and began to decrease by further increasing of nanotube length [29]. Kang and co-workers investigated the photoelectrochemical production of H_2_ from water splitting using CdS-sensitized Li-inserted TNTAs photoanode. H_2_ was generated at a faradaic efficiency of about 100% under irradiation with AM 1.5 light spectra. This superior performance was attributed to the effect of CdS sensitization and Li-ion insertion into TNTAs [30]. Zhang and co-workers successfully employed polylysine-modified TNTAs and Cu to design a pH-responsive drug release system which exhibited an enhanced control of the drug release rate at pH of 7.4 [31]. Free-standing TNTAs films were used to fabricate a fast-response and recovery H_2_S gas sensor [32]. Moreover, Gold nanoparticles-functionalized TNTAs, which exhibit localized surface plasmon resonance (LSPR) effect, were investigated in organic biomarker vapor sensing at room temperature and showed excellent photoelectrochemical amperometric detection capacity of volatile organic compounds associated with the disease, tuberculosis [33]. On the other hand, in spite of the above mentioned outstanding properties and many potential advanced applications of TNTAs, the widespread solar applications of TNTAs are hindered by the relatively wide energy band gap (E_g_) of TiO_2_ (3.2 eV for anatase and 3.0 for rutile) which is photoresponsive only to UV illumination. The visible light portion of the solar spectra is about 45% which is 9 times bigger than the UV portion (only 5%). Therefore, any enhancement in the photoactivity of TNTAs in the visible light region increases the harvesting power of solar energy and therefore has a positive impact on the diverse applications of this nanomaterial [34,35,36,37]. In order to achieve this objective and modify the properties of TiO_2_ to narrow its band gap and enhance the visible light responsiveness, metal ions such as Fe [38,39], Zr [40], Cr [41], and Zn [42] have been doped to TiO_2_ nanotube arrays using different doping techniques. Ag nanoparticles-sensitized TNTAs were recently prepared by Kong and co-workers. Ag nanoparticles could act as an electron-trap to minimize the recombination rate of photo-generated electron/hole pairs. Consequently, the photoelectrochemical and photocatalytic properties could be enhanced [43]. Recently, non-metals such as carbon [44], nitrogen [45,46,47], phosphorous-fluorine [48] and nitrogen-fluorine-iodine [49,50] have been doped into TiO_2_ nanotube arrays and proved significant enhancements in the visible light photoactivity. It is worth mentioning that doping of TNTAs is more difficult than TiO_2_ nanoparticles doping and presents a challenge as one should carefully choose the doping method that saves the nanotubular structure undamaged during the doping process. Particularly, carbon dopant has been introduced to TNTAs either by flame annealing [51], annealing under carbonaceous gas stream at elevated temperature [19] or hydrothermal treatment of TNTAs in an aqueous solution of glucose followed by annealing in Ar gas at 450 °C [52]. But these techniques either result in huge damage to the nanoarchitecture and crystal structure of the nanotube arrays [51] or involve complicated elevated temperature multistage processes [52,53,54].

Ordinary phases of the titania include amorphous, anatase and rutile. The crystal phase and nano-architecture of titania manipulate its properties and potential applications. For example, the anatase phase of titania is favored in catalysis and dye-sensitized solar cells applications, whereas rutile is preferred in the area of dielectrics and high-temperature oxygen gas sensors due to its excellent elevated temperature stability [24]. Moreover, the performance of TNTAs in different applications is highly dependent on the nanostructure parameters such as nanotube length, diameter and wall thickness. These parameters are mostly determined by adjusting the anodization conditions such as anodization potential, pH, anodization time and anodization electrolyte composition. Although a remarkable success has recently been achieved in fabrication of high-aspect-ratio TNTAs with nanotube length up to hundreds of micrometers, very little attention has been paid to study the effect of nanotube length on TNTAs performance, in particular, in the third generation of TNTAs with nanotube length longer than 20 µm [29,55].

In this study, we investigated a new facile and low-cost method to fabricate N- and C-modified TNTAs of length up to 55 µm by treating the as-anodized TNTAs in urea aqueous solution at room temperature for several hours. This method is also safe on the morphology and nanoarchitecture of TNTAs. The modified TNTAs were characterized by FESEM, X-ray diffraction, XPS, EDX and UV-Vis. diffuse reflectance spectra. Photoelectrochemical performance of modified and non-modified TNTAs was investigated. Moreover, we studied the effect of nanotube length on the photo-electrochemical performance.

## 2. Experimental Setup and Methods

### 2.1. Preparation of Modified TiO_2_ Nanotube Arrays (TNTAs)

The Ti foil (0.89 mm thickness, 99.6% purity) and all chemicals were purchased from Alfa-Aesar (Ward Hill, MA, USA). To prepare TNTAs, we used the same setup as in our previous work [56] and a modified procedure proposed by Prakasam and co-workers [3]. The Ti foil was cut into 1.5 cm diameter discs. Prior to anodization, the titanium foil was cleaned by using distilled water, acetone and distilled water, respectively, in ultrasonic bath for five minutes each. It was then dried off in air, etched in (3.3 M HF and 5.6 M HNO_3_) solution for 10 s and immediately rinsed with deionized water, dried with air and mounted into a Teflon electrode holder which allowed only 1 cm^2^ of one side of the disc to be exposed to the electrolyte while the whole backside area was isolated, then used in anodization process immediately. Ti foil was first anodized in ethylene glycol solution containing NH_4_F (0.4 wt %) and H_2_O (1 wt %) for 2 h at 60 V at room temperature using Keithley Source Meter Unit Model SMU 2602 (Keithley Instruments Inc., Cleveland, OH, USA). Ti foil was used as anode and a platinum foil was used as a cathode.

The produced nanotubes were removed by sonication in ethanol for 30 min to obtain textured fresh Ti surface. Subsequently, the second anodization was performed in the same electrolyte and the same anodization potential of 60 V. The second anodization time was changed to obtain different nanotube lengths. The anodization current was monitored with a computer. After the second anodization step, the samples were washed with distilled water and sonicated in water for 5 min to clean the surface and remove debris, then dried with air. The TNTAs samples were immersed in a 10 wt % urea solution and mechanically mixed for 30 min and kept immersed in urea solution for 6 h at room temperature to allow urea molecules to be adsorbed on TNTAs inner surface. Then annealed at temperature ranging from 350 to 650 °C for 3 h in air with a heating and cooling rate of 5 °C/min to promote crystallinity.

### 2.2. Characterization of Modified TNTAs

Titania nanotube arrays morphology was investigated with a field emission scanning electron microscope (FESEM, Hitachi S-5000, Tokyo, Japan) equipped with an energy dispersive X-ray analyzer unit (EDXA). The elemental compositions were determined by EDX analysis. The crystal structures were examined by X-ray diffraction using a powder X-ray diffractometer (Rigaku RINT 2500, Tokyo, Japan) with Cu K*α* radiation (*λ* = 1.54 Å) at 40 kV and 50 mA with a scan rate of 0.02^°^ per s over a 2*θ* range from 15° to 80°. The elemental chemical states were determined by X-ray photoelectron spectroscopy (XPS, Perkin Elmer, Waltham, MA, USA). The XPS analyses were carried out with a Kratos Axis Ultra spectrometer using a monochromatic Al K*α* source (15 mA, 14 kV). The instrument work function was calibrated to give a binding energy (BE) of 83.96 eV for the Au 4f7/2 line for metallic gold and the spectrometer dispersion was adjusted to give a BE of 932.62 eV for the Cu 2p3/2 line of metallic copper. The Kratos charge neutralizer system was used on all specimens. Survey scan analyses were carried out with an analysis area of 300 μm × 700 μm and a pass energy of 160 eV. High-resolution analyses were carried out with an analysis area of 300 μm × 700 μm and a pass energy of 20 eV. Spectra charge was corrected to the main line of the carbon 1s spectrum (adventitious carbon) set to 284.8 eV. CasaXPS software (version 2.3.14) was used to analyze the results. A Cary 100 UV-Vis-NIR spectrophotometer was used to measure the UV-Vis diffuse reflectance spectra.

### 2.3. Photoelectrochemical Properties

The photocurrent spectra were recorded by a home-made photoelectrochemical measurement system using an LPX150 Xe lamp solar simulator with a light intensity of 100 mW/cm^2^. The TNTAs sample served as the working electrode and a Pt sheet was used as the counter electrode. The testing electrolyte was 1 M KOH solution with 0.05 vol % ethylene glycol. A computer-controlled power supply (Keithley SMU 2602, Keithley Instruments Inc., Cleveland, OH, USA) was employed to control the potential and record the photocurrent generated. The light intensity at the quartz window of the electrochemical cell was fixed at 100 mW/cm^2^. The external potential was applied at a scan rate of 20 mV s^−1^ under illumination and the photocurrent was recorded. The potential of the open circuit was measured by a digital multimeter during the illumination.

## 3. Results and Discussion

### 3.1. FESEM and XRD Studies

**Figure 1:**
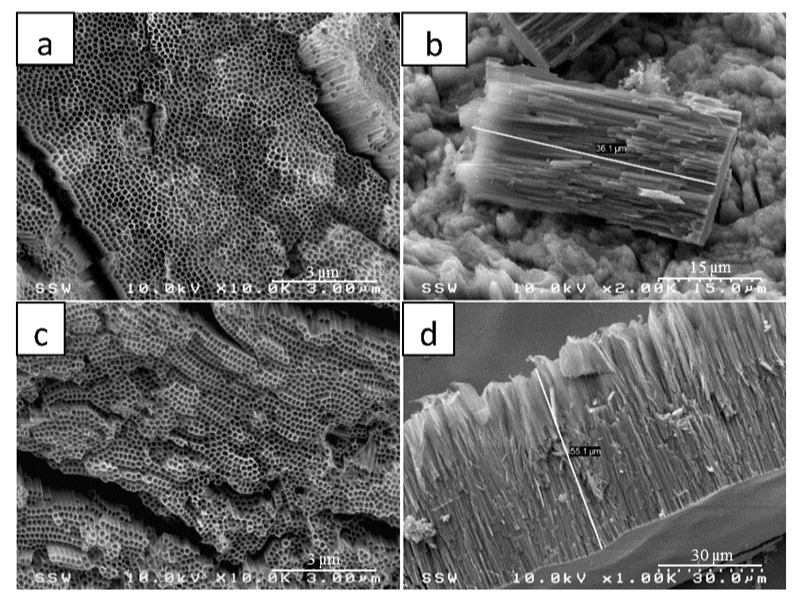

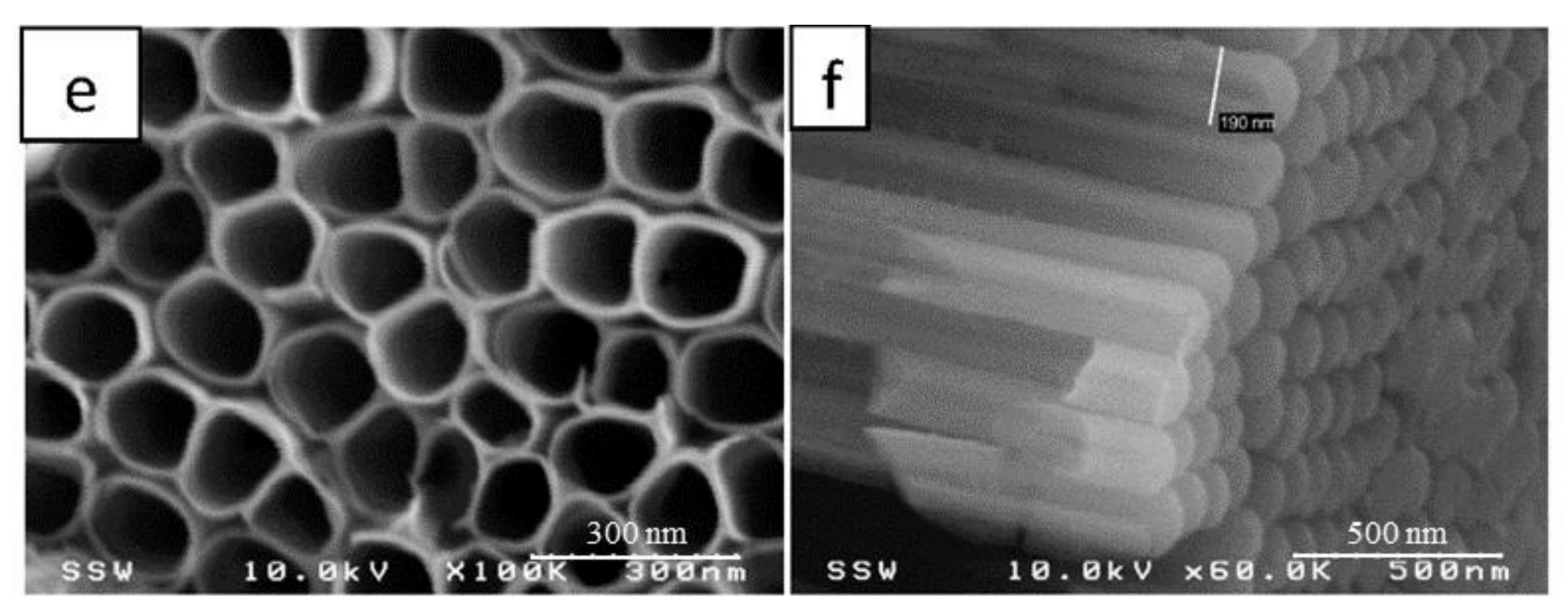
FESEM images for TNTAs synthesized in ethylene glycol electrolyte containing 1 wt % H_2_O and 0.4 wt % NH4F at constant potential of 60 V; (**a**,**b**) Tope view and cross sectional view of TNTAs anodized for 6 h and then sonicated in water for 5 min to clean surface and remove debris; (**c**,**d**) are top and cross sectional views of TNTAs anodized at the same conditions for 10 h; and (**e**,**f**) are high magnification of top and lateral views of TNTAs in (**a**), respectively.

Figure 1 shows the FESEM images of TNTAs fabricated in ethylene glycol electrolyte containing 1 vol % H_2_O and 0.4 wt % NH_4_F at 60 V. Figure 1a,b show the top view and the cross-sectional view of TNTAs anodized at room temperature for 6 h at room temperature, while Figure 1c,d represents top and cross-sectional views of TNTAs anodized in the same conditions for 10 h. The as-anodized TNTAs were sonicated in water for 5 min to clean surface and remove debris Figure 1e shows high magnification of top view and Figure 1f represents high magnification of cross sectional view near the TNTAs bottom showing the test-tube-like closed round bottom of the nanotubes. Clearly the smoothness of nanotube walls and the absence of ripples associated with the aqueous electrolytes anodization as in our previous work [56] are discernible. From the FESEM images, we conclude that TNTAs fabricated with this process are of high quality, well-organized, vertically oriented and homogeneous. The TNTAs are also reproducible as it can be seen by comparing the nano-architectures in the two samples shown in Figure 1a,c. The only difference is TNTAs length which increases by increasing the anodization time. The average inner diameter and wall thickness for TNTAs fabricated at 60 V in ethylene glycol are 173 ± 16 nm and 13 ± 1, respectively. The length of nanotube depends on the anodization time and is found to be 36 and 55 µm for 6 and 10 h anodization times, respectively (Figure 1b,d).

**Figure 2:**
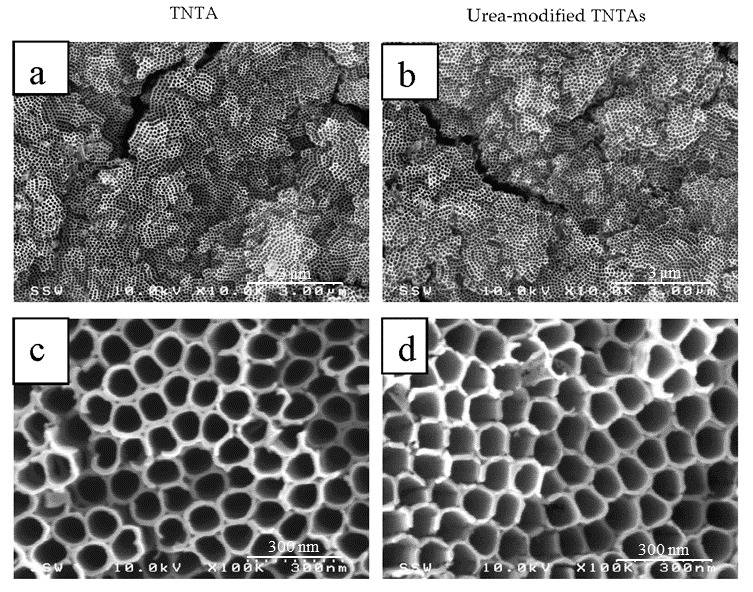
FESEM images of pristine (unmodified) and modified TNTAs: (**a**,**c**) top view and high magnified top view of pristine TNTAs; (**b**,**d**) top view and high magnified top view of modified TNTAs.

Ethylene glycol electrolyte helps not only in getting longer nanotubes in relatively short anodization time, but also in yielding smooth nanotube walls. One more difference between glycerol-water electrolyte and ethylene glycol electrolyte is the optimum anodization potential at which maximum nanotube growth rate can be achieved. The optimum anodization voltage for glycerol—water electrolyte was found to be 20 V [56], while it was 60 V for the ethylene glycol electrolyte [3]. Figure 2 shows the FESEM images of pristine TNTAs, Figure 2a,c (without modification), and Figure 2b,d the modified TNTAs. Both samples were annealed at 550°C for 3 h. As we can see from the FESEM images of pristine and modified TNTAs, the modification process did not cause any damage to the well-organized vertically oriented nanotubular architecture of TNTAs which is an important advantage of this modification method. In addition, the method is simple, low cost and environment-friendly.

**Figure 3:**
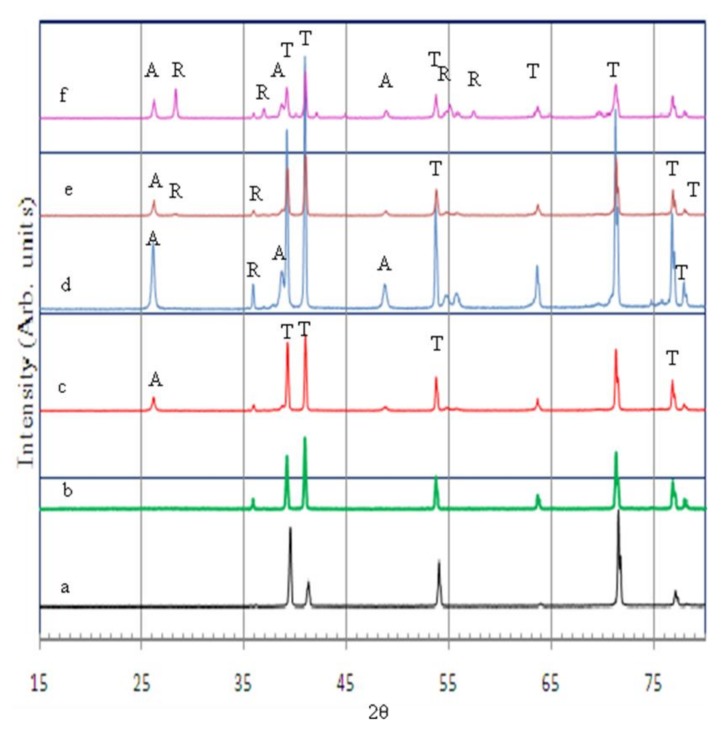
X-ray diffraction patterns for (**a**) Ti-metal foil; (**b**) as-anodized TNTAs; (**c**) TNTAs annealed at: 350 °C; (**d**) 450 °C; (**e**) 550 °C and (**f**) 650 °C, all for 3 h in air.

As-anodized TNTAs were amorphous and annealing at elevated temperature was required to promote crystallinity which increased the stability of the material and enhanced the charge transport and photoelectrochemical properties. By varying the annealing temperature, the phase structure of TNTAs can be controlled as anatase or rutile or a combination of both phases. As mentioned above, each crystal phase has its preferred application. Figure 3 shows the XRD patterns of TNTAs annealed at different temperatures from 350 to 650 °C for 3 h, as well as Ti-metal foil and as-anodized TNTAs for comparison. As it can be seen from Figure 3, both Ti-metal foil and as-anodized TNTAs (curves a and b, respectively) show only Ti metal peaks which implies that the as-anodized TNTAs layer was amorphous. Anatase phase began to appear at 350 °C (curve c) and increased and became dominant at 450 °C (curve d). At 450 °C, one can see traces of rutile phase. At 550 °C (curve e), the crystal structure of TNTAs was a conjugate of both anatase and rutile phases while the rutile phase became prominent when annealing at 650 °C (curve f). These X-ray results are consistent with other studies [16,57,58,59]. 

### 3.2. EDX and XPS Results

**Figure 4:**
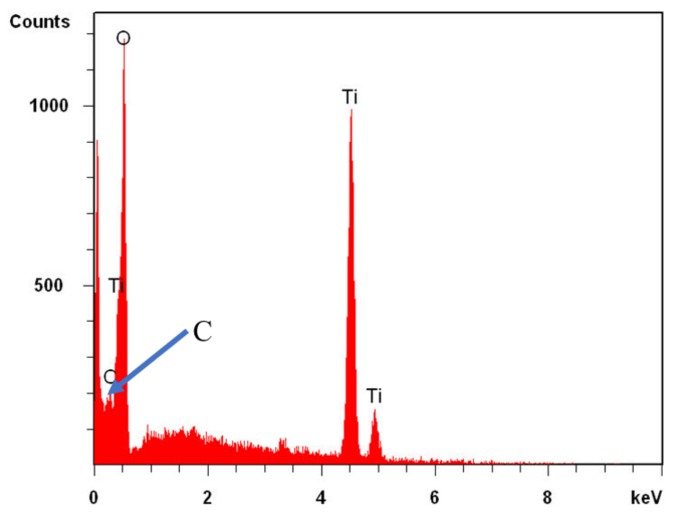
The EDX spectra of modified TNTAs indicating the elemental composition Ti and O with a ratio of Ti:O = 1:2 and the presence of carbon in TNTAs.

Elemental composition of TNTAs and determining whether foreign elements are introduced into TiO_2_ lattice can be investigated using both EDX and XPS spectra techniques. Figure 4 shows the EDX spectra of TiO_2_ nanotube arrays fabricated in ethylene glycol electrolyte and annealed at 550 °C for 3 h. The EDX spectra indicate the presence of Ti, O and C in the nanotubular sample and that the ratio of Ti:O is approximately 1:2 indicating that the structure of the nanotubes is TiO_2_ with the presence of carbon which comes from the organic electrolyte and urea after pyrolysis during annealing at elevated temperature. EDX analysis technique did not show nitrogen as it exists in low concentration that cannot be detected by EDX spectra.

**Figure 5:**
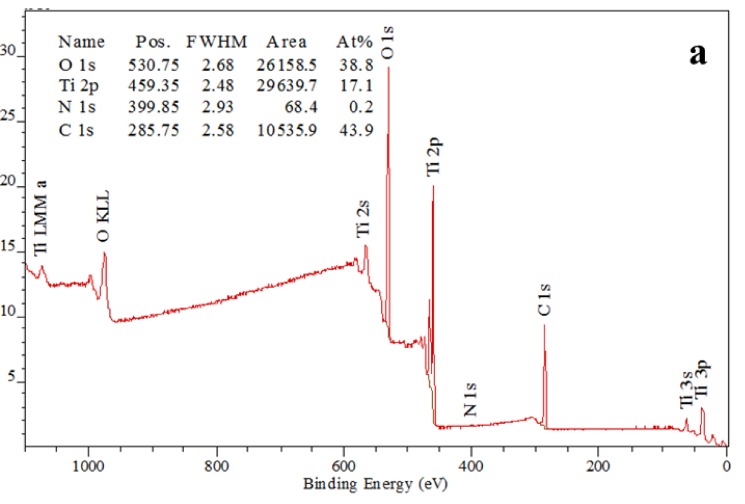

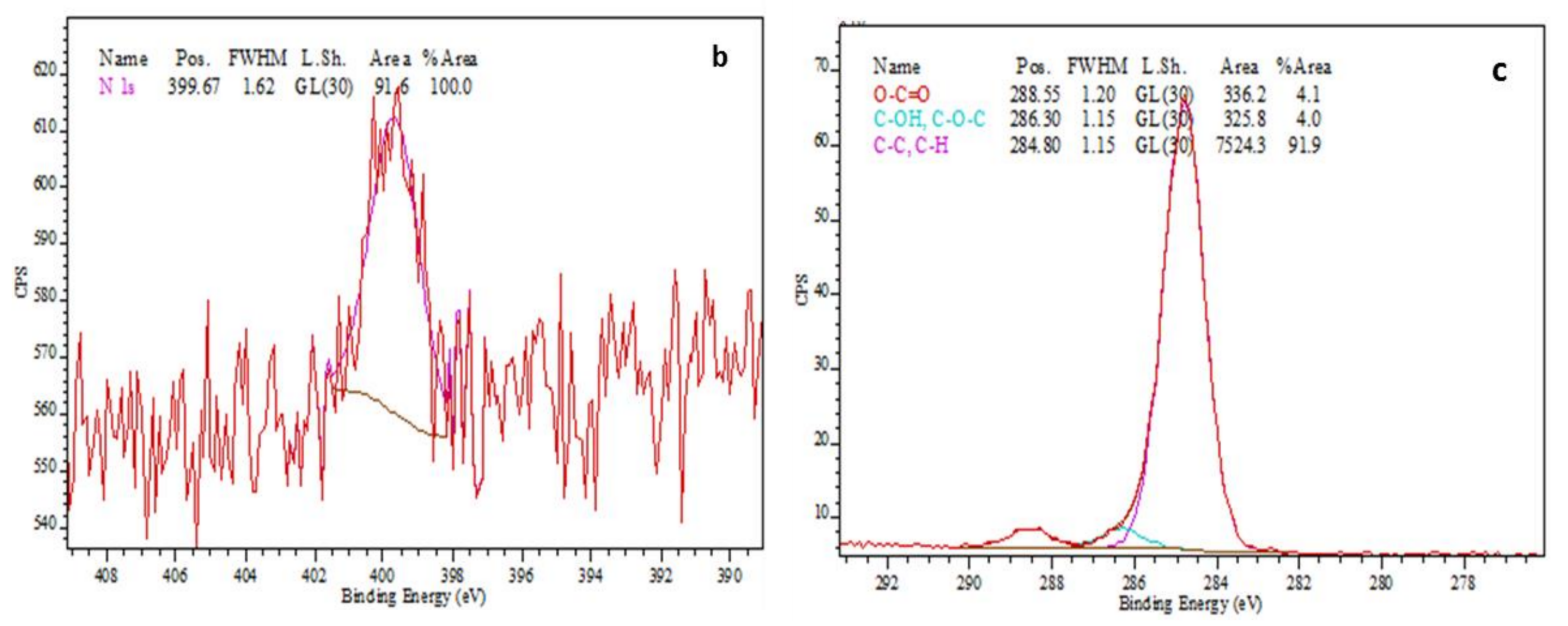
The XPS spectra of TiO_2_ nanotube arrays: (**a**) Wide range survey spectra; (**b**) High resolution XPS spectra over N 1s peak at 399.67 eV; and (**c**) High resolution XPS spectra over C 1s peak.

Figure 5 shows the XPS spectra for the TNTAs sample annealed at 550 °C for 3 h in air. As it can be seen from Figure 5a, TNTAs contained Ti, O, C and N. The binding energies of Ti 2p, O 1s, C 1s and N 1s were 459.35, 530.75, 285.75 and 399.67 eV, respectively. The atomic ratio of Ti:O was very close to 1:2 implying that the chemical composition of nanotubes was TiO_2_. Figure 5b shows high resolution of N 1s peak at binding energy of 399.67 eV. This binding energy exists between two binding energies at 400.1 and 398.2 eV which correspond to C-N=C and C-NH_2_, respectively [60]. This suggests that the existing nitrogen is mainly bonded to carbon atoms. The high resolution of C 1s peak region (Figure 5c) exhibits the following peaks; the peak at 284.8 eV is assigned to adventitious carbon, and the peak at 286.3 eV is ascribed to C=O bonds due to pyrolysis of urea during annealing [61], and the peak at 288.55 eV is attributed to the carbon existing in the form of interstitial atoms which takes place due to the diffusion of carbon atoms during the annealing process at elevated temperature [19]. The XPS spectra proved that N atoms existed as pyrolyzed urea on TNTAs surface while C atoms existed as pyrolyzed urea products on the surface as well as interstitial atoms diffused into the crystal structure of TNTAs. 

### 3.3. Optical Properties

**Figure 6:**
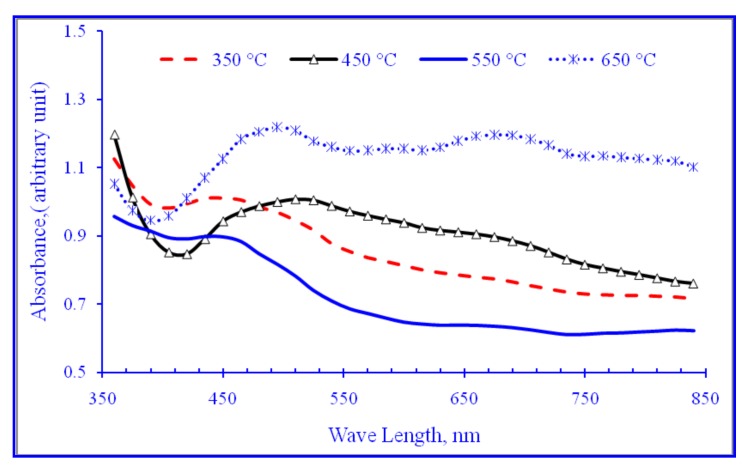
UV-Vis diffuse reflectance absorbance of TiO_2_ annealed at different temperatures.

**Figure 7:**
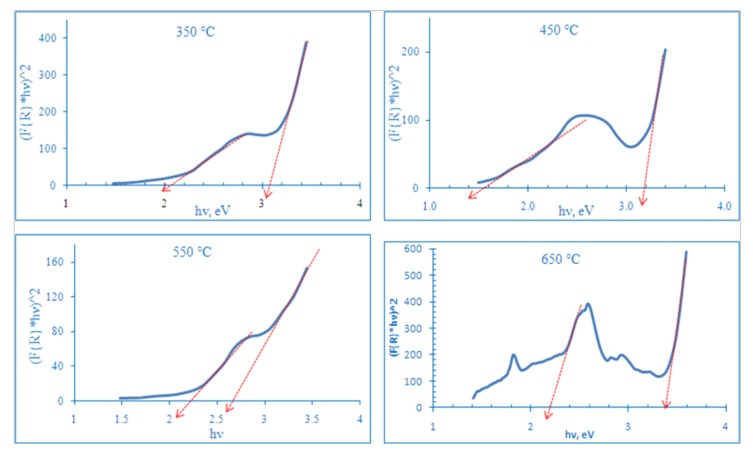
Kubelka-Munk transferred diffuse reflectance spectra of samples annealed at 350, 450, 550 and 650 °C. The intersections of red rows with X-axis represent the values of band gap energy.

The UV-Vis diffuse reflectance absorption spectra of modified TNTAs as a function of annealing temperature are shown in Figure 6. As it can be seen easily from Figure 6, annealing temperature has a large effect on the light absorbance of the nanotube arrays because annealing temperature not only affects on the crystallization process but also the incorporation of foreign elements such as carbon and nitrogen into TiO_2_ crystal lattice. Figure 6 indicates that all samples have strong absorbance in the visible region with the best absorbance for the sample annealed at 650 °C. In Figure 7, absorbance data from Figure 6 were manipulated using Kubelka-Munk equation to calculate the modified band gap energies for each sample [62].

**Figure 8:**
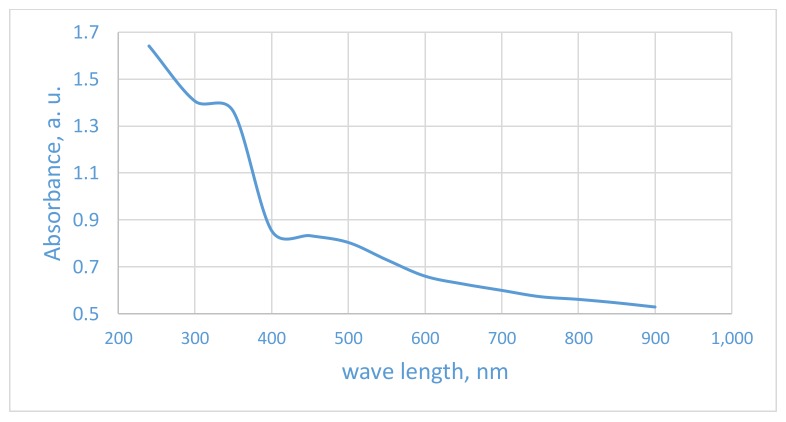
UV-Vis diffuse reflectance absorbance of pristine (pure) TiO_2_ nanotube arrays annealed at 550 °C.

From Figure 7 and Table 1, each sample has two band gap energies, a primary one and a secondary one due to the effect of the electronic levels of the foreign dopants. The primary one has the larger effect on photoelectrochemical properties of TNTAs. As seen in Figure 7 and in Table 1, the sample annealed at 550 °C has the smallest primary band gap energy, E_g_ (2.65 eV), which corresponds to the absorbance edge wavelength of 468 nm while sample annealed at 450 °C has the smallest secondary band gap energy, E_g_ (1.6 eV), which corresponds to absorbance edge wave length of 775 nm. The slight change in absorbance and band gap energy of samples annealed at 350 and 450 °C can be attributed to the surface impurities due to pyrolyzed urea products and partial crystallization of TNTAs. While the substantial change of visible light absorbance and band gap energy in samples annealed at 550 and 650 °C can be attributed to the N and C modification of TNTAs surface and interstitial C atoms as shown by XPS spectra. Pure (unmodified) TNTAs showed only UV absorbance with absorbance edge at 400 nm (band gap energy: 3.1 eV) as shown in Figure 8. 

### 3.4. Photoelectrochemical Properties

**Figure 9:**
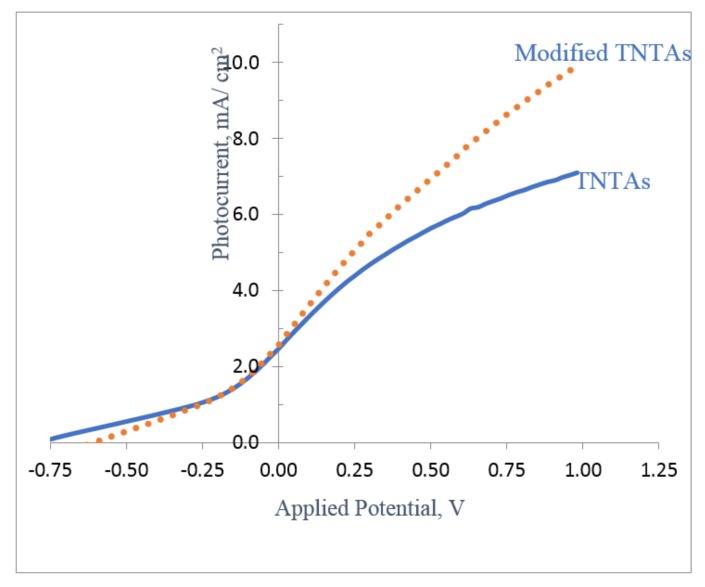
Photocurrent density of modified TNTAs compared with that of pristine (unmodified) TNTAs. Both samples were anodized in the same conditions with identical nanoarchitectures.

Figure 9 shows photocurrent density, I_ph_, of modified TNTAs compared with that of (unmodified) TNTAs. Both samples were anodized and treated at the same conditions to obtain identical nanoarchitectures. Unmodified (pristine) sample was annealed directly at 550 °C for 3 h while the other sample was first treated with urea solution as described in the experimental part, then annealed at 550 °C for 3 h. Both samples have nanotube length of 25 µm, an inner diameter and a wall thickness of 173 ± 16 and 13 ± 1 nm, respectively. As we can see from Figure 9, the modified TNTAs shows a considerable increase in I_ph_ compared with the pristine (unmodified) TNTAs. I_ph_ increased from 7.1 mA/cm^2^ for pristine (unmodified) TNTAs to 9.9 mA/cm^2^ for modified TNTAs with a maximum % increase of 39% at 1 V applied potential. 

**Figure 10:**
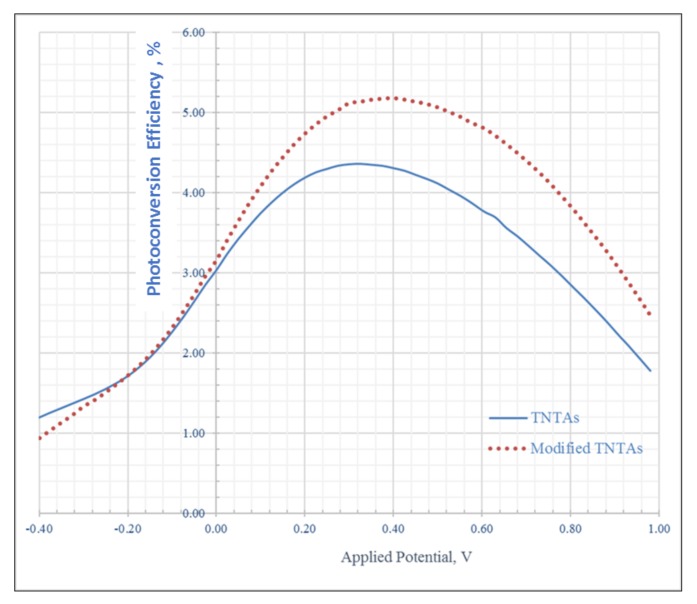
Photoconversion efficiency of modified TNTAs and pristine (unmodified) TNTAs.

Figure 10 shows the % photoconversion efficiency, PCE, for modified and unmodified TNTAs as a function of applied potential. The percent PCE was calculated using the following equation [18,63]:

PCE (%) = [I_ph_ × (1.23 − E_app_) × 100]/I_0_ (1)

E_app_ = E_meas_ − E_oc_ (2)

where I_ph_ is the photocurrent density in mA/cm^2^, E_app_ is the external applied potential in V given in Equation (2), E_meas_ is the measured bias potential in V (vs. Ag/AgCl reference electrode), and E_oc_ is the electrode potential (vs. Ag/AgCl) of the same working electrode at open circuit conditions under the same illumination and in the same electrolyte. As we can see from Figure 9, the photoconversion efficiency, was increased from 4.35% for pristine (pure) TNTAs to 5.18% for modified TNTAs with an increase of 19%. The high value of photocurrent density and photoconversion efficiency of both pristine and modified TNTAs measured in this work was attributed to the high surface area of long nanotubes (25 µm) and the presence of 0.05% ethylene glycol as electron donor to decrease the charges recombination rate.

Although vast number of studies have been conducted in the last decade on fabrication of long TNTAs of up to hundreds of micrometers [64,65], there is a shortage of studies of photoelectrochemical performance of long nanotubes. The increase in the surface area by increasing nanotubes length enhances the capacity to harvest light photons. But on the other hand, diffusion path of the electrons to reach the conducting layer in the back of the photoanode increases with increasing the nanotubes length which results in increasing the charge recombination rate. A series of TNTAs samples were fabricated at the same anodization conditions mentioned above. Only anodization time was changed from 3 h to 10 h to produce TNTAs with the same inner diameter and wall thickness but with different nanotube lengths as shown in Table 2. All TNTAs samples were treated with urea solution as described in the experimental part to produce C- and N-modified TNTAs then annealed for 3 h at 550 °C.

**Figure 11:**
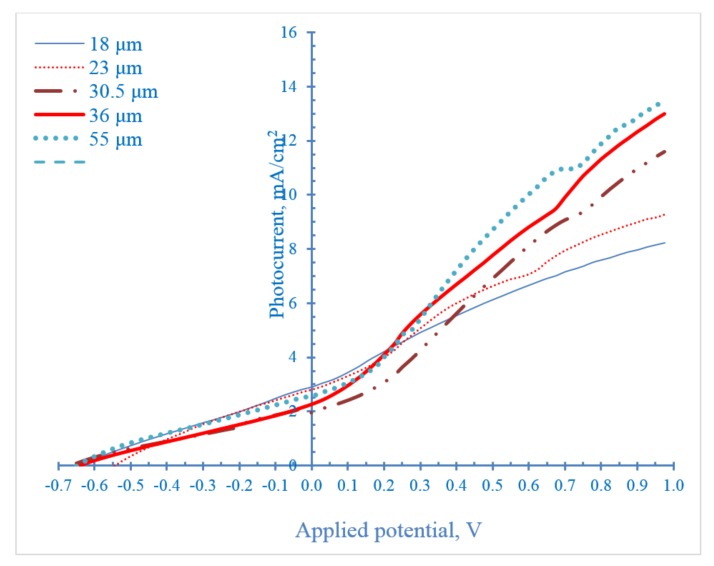
Effect of nanotubes length on photocurrent as a function of applied potential.

Figure 11 shows the photocurrent density of modified TNTAs with different lengths as a function of applied potential. As we can see, photocurrent increases with increasing nanotubes length. This implies that due to the one dimensional vertically oriented nanotubular architecture of the photoanode, the light photons successfully penetrated the entire length of the nanotube arrays up to 55 µm and the huge surface area increased the light harvesting capacity of TNTAs. Furthermore, the presence of ethylene glycol as electrons donor, although in very small concentration (0.05 vol %), reduced the charge recombination rate in very long nanotubes photoanode.

**Figure 12:**
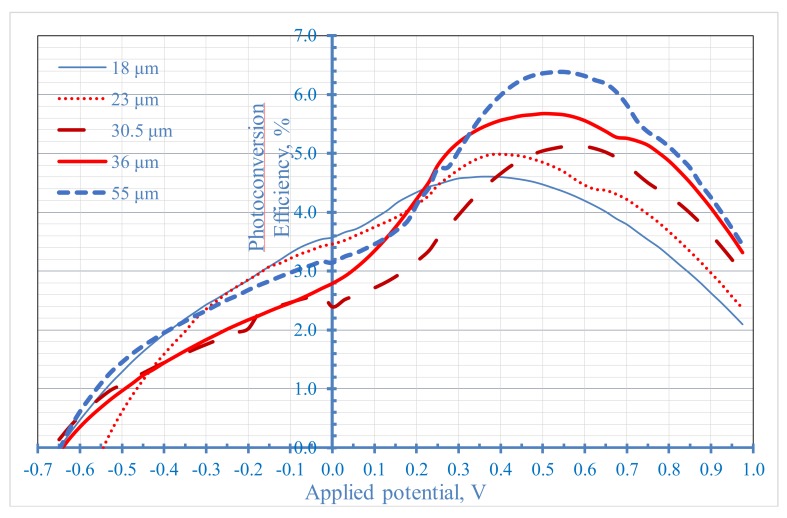
Effect of nanotube length on photoconversion Efficiency, PCE, as a function of applied potential.

**Figure 13:**
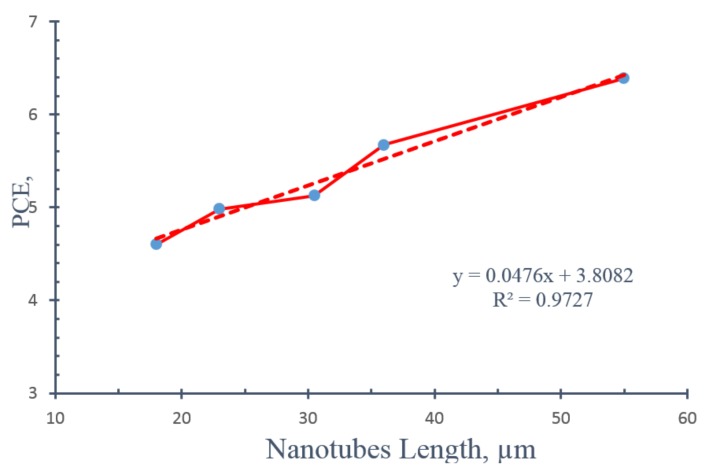
Linear dependence of photoconversion efficiency on nanotube length of modified TNTAs in the range from 18 to 55 µm. The solid line represents the actual experimental data and the dotted line represents the best-fitting straight line through the data which is defined by the equation shown in the figure.

Effect of nanotube length on photoconversion efficiency, PCE, is shown in Figure 12. Photoconversion efficiency increases with increasing nanotube length with a maximum PCE of 6.38% at 55 µm nanotubes length and applied potential of 0.55 V. It should be noted that the applied potential at which maximum PCE was obtained increased from 0.35 V at nanotube length of 18 µm to 0.55 V at nanotube length of 55 µm as can be seen in Figure 12. Figure 13 shows a linear relation between maximum PCE of each sample and the nanotube length of the same sample in the range from 18 to 55 µm. The increase of maximum PCE with increasing nanotube length and the linear dependence of maximum PCE on nanotube length up to 55 µm imply that the light successfully penetrates the long TNTAs photoanode up to 55 µm which is more than 3.6 times longer than the maximum layer thickness of the nanoparticulate photoanode that light can penetrate (15 µm).

## 4. Conclusions

In this investigation, a new, facile, low cost and environmental-friendly method for the fabrication of N- and C-modified TiO_2_ nanotube arrays was reported. The titania nanotube arrays were immersed in a 10 wt % urea solution with mechanical agitation for 30 min. The TNTAs were immersed in the solution for 6 h at room temperature. Then, TNTAs were annealed at different temperatures. Modified TiO_2_ nanotube arrays with different lengths from 18 µm to 55 µm were synthesized in this study. The produced N- and C-modified TNTAs were characterized by FESEM, EDX, XRD, XPS, UV-Vis diffuse reflectance. Modified optical properties with narrow band gap energy, E_g_, of 2.65 eV was obtained after annealing the modified TNTAs at 550 °C. Modified TNTAs showed enhanced photocurrent density and photoconversion efficiency. Photoconversion efficiency, PCE, was increased from 4.35% for pristine (unmodified) TNTAs to 5.18% for modified TNTAs, an increase of 19%. Effect of nanotubes length of modified TNTAs on photoelectrochemical performance was studied. Photoconversion efficiency PCE was increased by increasing nanotube length with maximum PCE of 6.38% at nanotube length of 55 µm. The PCE increase pattern was linear with nanotubes length. This implies excellent light penetration up to 55 µm depth into photoanode which is 3.6 times higher than the maximum penetration depth (15 µm) in the nanoparticulate photoanode. This increasing pattern of photoconversion efficiency with increasing nanotubes length also implied a high charge separation rate and lower charge recombination rate. The high PCE was attributed to band gap reduction due to C- and N-modification of TNTAs and the increased surface area of long TNTAs compared to short TNTAs resulted in excellent light penetration and harvesting properties. 

## Figures and Tables

**Table 1:** The band gap energies (E_g_) and corresponding absorbance edges of TiO_2_ nanotube arrays annealed at different temperatures.

Anneal. Temperature	Main E_g_, eV	Secondary E_g_, eV	Main Abs. Edge, nm	Secondary Abs. Edge, nm
350 °C	3.1	2.1	400	590
450 °C	3.2	1.6	387	775
550 °C	2.65	2.2	468	564
650 °C	3.38	2.2	367	564

**Table 2:** Nanotubes lengths anodized for different anodization times.

Anodization Time, h	3	4	6	7	10
Nanotubes Length, µm	18	23	30.5	36	55
